# Dive Risk Factors, Gas Bubble Formation, and Decompression Illness in Recreational SCUBA Diving: Analysis of DAN Europe DSL Data Base

**DOI:** 10.3389/fpsyg.2017.01587

**Published:** 2017-09-19

**Authors:** Danilo Cialoni, Massimo Pieri, Costantino Balestra, Alessandro Marroni

**Affiliations:** ^1^DAN Europe Research Division, DAN Europe Foundation Roseto degli Abruzzi, Italy; ^2^Environmental, Occupational and Ageing (Integrative) Physiology Lab, Haute Ecole Bruxelles-Brabant Brussels, Belgium

**Keywords:** SCUBA diving, decompression sickness, decompression illness, vascular gas emboli, decompression algorithms

## Abstract

**Introduction:** The popularity of SCUBA diving is steadily increasing together with the number of dives and correlated diseases per year. The rules that govern correct decompression procedures are considered well known even if the majority of Decompression Sickness (DCS) cases are considered unexpected confirming a bias in the “mathematical ability” to predict DCS by the current algorithms. Furthermore, little is still known about diving risk factors and any individual predisposition to DCS. This study provides an in-depth epidemiological analysis of the diving community, to include additional risk factors correlated with the development of circulating bubbles and DCS.

**Materials and Methods:** An originally developed database (DAN DB) including specific questionnaires for data collection allowed the statistical analysis of 39,099 electronically recorded open circuit dives made by 2,629 European divers (2,189 males 83.3%, 440 females 16.7%) over 5 years. The same dive parameters and risk factors were investigated also in 970 out of the 39,099 collected dives investigated for bubble formation, by 1-min precordial Doppler, and in 320 sea-level dives followed by DCS symptoms.

**Results:** Mean depth and GF high of all the recorded dives were 27.1 m, and 0.66, respectively; the average ascent speed was lower than the currently recommended “safe” one (9–10 m/min). We found statistically significant relationships between higher bubble grades and BMI, fat mass, age, and diving exposure. Regarding incidence of DCS, we identified additional non-bubble related risk factors, which appear significantly related to a higher DCS incidence, namely: gender, strong current, heavy exercise, and workload during diving. We found that the majority of the recorded DCS cases were not predicted by the adopted decompression algorithm and would have therefore been defined as “undeserved.”

**Conclusion:** The DAN DB analysis shows that most dives were made in a “safe zone,” even if data show an evident “gray area” in the “mathematical” ability to predict DCS by the current algorithms. Some other risk factors seem to influence the possibility to develop DCS, irrespective of their effect on bubble formation, thus suggesting the existence of some factors influencing or enhancing the effects of bubbles.

## Introduction

The popularity of SCUBA diving is steadily increasing together with the number of dives and correlated diseases per year, even if the total number of exposed individuals (i.e., commercial divers, hyperbaric attendants, and recreational divers) and the exact incidence of decompression illness DCI is unknown (Trout et al., 2012).

This pathology is affecting divers, astronauts, pilots, and compressed air workers, and although its occurrence is relatively rare, with rates of 0.01–0.1% per dive (the higher end of the spectrum reflecting rates for commercial diving and the lower rates for scientific and recreational diving), the consequences can be dramatic (Balestra et al., 2016).

The increase in ambient pressure, the different breathing gases used for diving (with different fractions of inert, saturating gases), the rules that govern their behavior and the correct decompression procedures are considered well known (Bennett and Elliott, 1982). Commonly, decompression tables or diving computers are used to control the risk of decompression sickness (DCS) using the “leading tissue” concept to calculate decompression stop depth and time (Buhlmann, 1982).

In Haldane-Bulhmann decompression models, for instance, the decompression algorithm is calculated not to exceed a given maximum inert gas level, for each “compartment,” the so called: *M*-value (Buhlmann, 1982).

Even if the ultimate pathogenic mechanism of DCS is still debated, the link between circulating inert gas emboli (Vascular Gas Emboli: VGE) and DCS is well accepted, as well as the presence of “silent” VGE in many divers without any DCS symptom (Weathersby et al., 1987; Eftedal et al., 2007).

New recently developed hypotheses indicating that inert gas embolism can trigger cell-mediated mechanisms assimilating DCS to an inflammatory disease (Thom et al., 2015) make the presence of even “silent bubbles” worth considering and investigating to identify further risk factors that may correlate with an increase in the incidence of bubble formation and DCS.

Our hypothesis may also encompass that some predisposing factors and/or peripheral humoral variables can contribute to develop DCS at the same level of bubble degree.

This study aims at three goals:

1. An in-depth epidemiological analysis focusing on habits and risks of the diving community.
2. Investigating additional risk factors correlated with the development of circulating bubbles other than pressure differentials.
3. Analyzing 320 DCS cases from the DAN Europe Diving Safety Laboratory (DSL) database (DAN DB) to identify related risk factors and to improve the current decompression guidelines.

## Materials and methods

An original database (DAN DB) including specific questionnaires for data collection was developed allowing retrospective statistical analysis from 2,629 European divers (2,189 men 83.3%, 440 women 16.7%) who made 39,099 open circuit dives over 5 years. All dives were considered started upon reaching 1 m depth and finished when reaching the same depth without any return to deeper depth within 5 min.

All dives shallower than 5 m and shorter than 10 min were excluded.

### Description of the SCUBA community and dives

Information about gender, age, and anthropometric data (height, weight) were requested, BMI was calculated. Starting from age, gender and BMI we also extrapolated the percentage of fat mass and lean body weight, using the Deurenberg (Deurenberg et al., 1991a,b) and the James formula, respectively (James and Waterlow, 1976).

All the 39,099 dives have been digitally recorded including depth, diving time, relative gradient factor (GF), and real water temperature.

The maximum gradient factor (GF) was calculated according to the Buhlmann ZHL16 C model, taking into account any previous dive if the surface interval was less than 48 hours (repetitive dives). Dives made after a surface interval longer than 48 hours were considered as non-repetitive.

GF is a way to measure nitrogen supersaturation in the “leading tissue” (the compartment with the highest supersaturation level) at any given time and depth during the ascent to the surface, represented as a fraction of the maximum inert gas supersaturation (*M*-value) allowed for the 16 tissues considered by the Buhlmann ZH-16 Model C, from 4 to 635 min half saturation/desaturation times (HT). Calculations of GF were performed for all the 16 tissues, and the maximum GF-value in the leading tissue was recorded (Baker, 1998).

The frequency of leading tissue involvement was also investigated. Aqueous tissues, with low gas solubility, were usually considered “fast tissues” (in terms of saturation time) as compared to “slow tissues” with high gas solubility (Bennett and Elliott, 1982; Marroni et al., 2004); starting from this the various “tissues” were grouped into three Leading Tissue Groups (LTG) in the DAN DB, to better manage the 16 investigated tissues, data recording and the related statistical analysis:

✓ Fast tissues (from 4 to 18.5 HT)
✓ Medium tissues (from 27 to 38.3 HT)
✓ Slow tissues (from 54.3 to 635 HT)

The dive profiles were collected from different models of diving computers and converted into the original DAN DB format known as DAN DL7 allowing us to recalculate the dive profile in an extended way that permits a detailed analysis with different calculations based on “real time” depth and time data points.

Trimix dives (open circuit, semi closed and closed circuit) were excluded.

A specific questionnaire was used to also record other dive characteristics such as:

✓ Environment (sea, lake, other)
✓ Purpose of diving (sightseeing, research, learning, photography, other)
✓ Gas used (air or nitrox)
✓ Current (absent or present)
✓ Visibility (low < 7/10 m; high >7/10 m)
✓ Perceived individual thermal comfort
✓ Type of diving suit (dry or wet)
✓ Physical condition before the dive (rested or tired)
✓ Level of exercise during the 24 h preceding diving (no-exercise, moderate or heavy exercise)
✓ Workload during the dive (no-workload, light or intense)
✓ Diver related problems during diving (difficult ear equalization, out of air, buoyancy control, shared air, rapid ascent, omitted deco, vertigo, seasickness, other)
✓ Equipment problems (jacket, regulator, dive computer, mask, fins, suit, weight belt, other)
✓ Use of alcohol
✓ Use of “habitual” drugs (medically prescribed) during the 24 h before diving
✓ Use of “occasional” drugs (non-medically prescribed) during the 24 h before diving

Additional information about general medical history (allergy, asthma, heart, and vascular disease, pulmonary problems, diabetes, recurrent back pain, sinus problems, previous DCS, and use of tobacco were also requested.

### Bubble formation risk

Nine hundred and seventy out of the 39099 collected dives were also investigated for bubble formation by 1-min precordial Doppler recording at 30 min after surfacing, 448 out of 970 were also recorded every 15 min and for 90 min after surfacing.

Doppler recordings were evaluated according to a modified Spencer Scale (Spencer and Johanson, 1974) named Expanded Spencer Scale (ESS) (Marroni et al., 2004) as follows:

**Table:** 

Grade 0	No Bubble Signals
Grade 0.5	1–2 sporadic Bubble signals
Grade 1	up to 5 Bubble signals
Grade 1.5	up to 15 Bubble signals
Grade 2	up to 30 Bubble signals
Grade 2.5	more than 30 Bubble signals
Grade 3	virtually continuous Bubble signals
Grade 3.5	continuous Bubble signals, with numerous bubble showers
Grade 4	continuous Bubble signals, with continuous bubble showers.

However a simplified bubble grading system was used for our statistical evaluation, as follows: (Marroni et al., 2004)

**Table:** 

Zero	No Bubble signal
LBG	Low Bubble Grade: occasional bubble signals, lower than 2 in the ESS
HBG	HBG High Bubble Grade: Frequent to continuous bubble signals, 2 and 2.5 in the ESS
HBG+	High Bubble Grade plus: Bubble signals reaching grade 3, 3.5, and 4 in the ESS.

Bubble grade was compared with several parameters and possible risk factors such:

✓ Gender and age
✓ Height
✓ Weight
✓ BMI
✓ Fat mass and lean body weight
✓ Diving profile (depth, time, GF, and LTG)
✓ Minimum water temperature
✓ Environment
✓ Purpose of diving
✓ Gas used
✓ Current
✓ Visibility
✓ Perceived individual thermal-comfort
✓ Type of diving suit
✓ Physical conditions before dives
✓ Exercise during the 24 h preceding diving
✓ Workload during the dive
✓ Any diver and equipment related problem during diving
✓ Use of alcohol

### DCS associated risk factors

To better understand the mechanisms of DCS, we also comparatively analyzed 320 sea-level dives followed by DCS symptoms.

These DCS cases were evaluated according to the same dive parameters and risk factors investigated for bubble formation and compared to the no-DCS dives in the DAN DB.

We also made an in-depth analysis of GF and LTG distribution in the DCS cases.

## Statistical analysis

Data are presented as the mean ± standard deviation (SD) for parametric data and median and range for non-parametric data (e.g., bubble grades).

The risk factors related effect in bubbles formations were investigated by non-parametric analysis of variance (Kruskal–Wallis test), after normality testing (Kolmogorov–Smirnov test) for anthropometric and diving data and by the chi-square test for gender and environmental data, leading tissue, and for the other risk factors.

The influences of the risk factors in the development of DCS were compared with the no-symptomatic dives in the DB by the Mann-Whitney test for non- parametric data, after the normality test (Kolmogorov-Smirnov) for anthropometric, diving and other risk factors data. Some data such as: gender, visibility, current, environmental, exercise before diving, state before, thermal comfort, use of alcohol, diver, and equipment problem were investigated using the chi-square test.

## Results

### Description of the SCUBA community and dives (Table 1)

Two thousand six hundred and twenty-nine divers (2,189 male, 440 female; mean age 37.36 ± 9.17 years) completed 39,099 open circuit dives (32,311 – 82.6% performed by males and 6,788 – 17.4% performed by females); mean age (mean ± SD) was 37.4 ± 9.2 years (38 for males and 34 for females).

**Table 1:** Description of the SCUBA community.

**Anthropometric data**	**Male**	**Female**	**Total**
**Gender**	***N*** = **2,189**	***N*** = **440**	***N*** = **2,629**
	**83.3%**	**16.7%**	
Age (years)	38	34	37.4 ± 9.2
Height (cm)	177	164	175.3 cm ± 6.22
Weight (km)	81	61	77.6 Kg ± 9.27
BMI (kg/m^2^)	26	23	25.16 ± 1.83
Fat mass (%)	23.2	29.4	23.7% (6.5–43.6)
Lean body weight (%)	62.6	44.8	62.6 (34.3–87.3)
**CHARACTERISTIC OF DIVES**			
Diving profile	27.1 (5–104) depth	46.4 (10–130) diving Time	0.66 (0.05–1.25) GF
Leading tissues	70% medium	24.5 fast	5.5% slow
Real temperature recorded	17.28°C (±6.53)		
**RISK FACTORS**			
Environment (Lake/Sea)	86.1% seawater	6.2% lake	7.7% other
Scope	66.2% sightseeing	11.2% research	22.6% other
Gas used	95.3 air	4.7 nitrox	
Current	77.3 absent	22.7 present	–
Visibility	33.3 < of 7/10 m	66.7 >of 7/10 m	–
Perceived temperature-comfort	94.7% thermal comfort	5.3% “felt cold”	
Suit	60.5% wet	19.0% dry	20.6% not filled
Feeling before the dive	90.9% rested	9.1% tired	–
Physical exercise during the 24 h before dive	30.3% no exercise	68.8% moderate	0.9% heavy
Workload during the dives	92.1% no-workload or light	7.9% intense	–
Diver related problems	96.4 no problem	1.25 equalization 0.45 out of air 0.42 Buoyancy	0.05 omiss. Deco 0.28 rapid ascent 1.19 Other
Equipment problems	97.2 no problem	0.29 Jacket 0.26 Octopus 0.10 Computer 0.83 Mask	0.08 Fins 0.40 Suit 0.32 Weight belt 0.47 Others
Moderate use of Alcohol before	57.6 no use	42.4 moderate use	–
Use of drugs	2.6% habitual drugs (Prescribed)	1.2% occasional drugs (not prescribed)	3.8% total
Medical history	12% at least one chronic diseases	14.7% smoker	1.1% previous DCS

Anthropometric and diving profile data were as follows:

✓ Mean height 175.3 cm ± 6.22 (177 males–164 females)
✓ Weight 77.6 Kg ± 9.27 (81 males–61 females)
✓ BMI 25.16 (Kg/m^2^) ±1.83 (26 males–23 females)
✓ Fat mass calculated 23.7% range 6.5–43.6 (23.2 males–29.4 females)
✓ Lean body weight 62.6% range 34.3–87.3 (62.6 males–44.8 females)
✓ Depth 27.1 m (range 5–104); Dive time 46.4 min (range 10–130); GF 0.66 (range 0.05–1.25)
✓ Half Time grouping: medium tissue 70.0%; fast tissue 24.5%; slow tissue 5.5% of cases
✓ Mean water temperature 17.28°C (SD ± 6.53)
✓ All dives were performed with open circuit SCUBA (95.3% Air; 4.7% Nitrox)

The other characteristics recorded by the questionnaire were:

✓ Dives in seawater 86.1%, in lake 6.2% and in different conditions 7.7%
✓ 66.2% were made for sightseeing, 11.2% for research, 6.1% for learning, 2.6% for photography, and 13.9% for other scope
✓ Current was reported as absent in 77.3% and present in 22.7%
✓ Visibility was reported as low in 33.3%, and high in 66.7% of cases (Data about current and visibility were filled only for 14,361 dives)
✓ 5.3% declared to have “felt cold” while 94.7% reported thermal comfort during the dive
✓ Wet suits were used in 60.5% of the dives, dry suits in 19.0% (in 20.6% of dives this field has not been filled)
✓ 90.9% of the divers declared being rested, while 9.1% declared being tired before the dive
✓ 30.3% declared no exercise, 68.8% moderate exercise and 0.9% heavy exercise during the pre-dive 24 h period
✓ Reported Workload during the dive: No or light-workload 92.1%, intense 7.9%
✓ Diver related problem during diving were reported by 3.6% while 96.4% declared no problem (details in Table 1)
✓ Equipment malfunction during diving occurred in 2.7% of the dives (details in Table 1)
✓ 42.4% of the divers declared moderate alcohol use and 57.6% no alcohol use in the 24 h before diving
✓ In 1,010 dives out of 39,099 (2.6%), divers declared use of “habitual” drugs (medically prescribed) and 490 (1.2%) use of occasional (non-medically prescribed) drugs; a total of 1,500 dives (3.8%) were performed after some drug use
✓ 316 subjects (12%) declared at the least one chronic disease; 386 were smokers (14.7%) and 28 had suffered previous DCS (1.1%)

### Bubble formation risk (Table 2)

Nine hundred and seventy (892 male and 78 female) precordial Doppler files were evaluated according to the ESS scale and converted into the simplified Doppler grading system: 369 dives (38%) showed no bubble, 446 dives (46%) Low Grade Bubbles (LBG), 106 dives (11%) High Grade Bubbles (HBG), and 49 dives (5%) showed High Grade Bubbles Plus (HBG+).

Table 2Bubble formation Risk: Only Age and BMI influence bubble formation, all the other investigated risk factors did not show any effect on bubble formation or had an influence BUT trough modification of diving exposure.

**Table:** 

**Sample description**				
Gender	*N* = 892 male	*N* = 78 female	*N* = 970 total	
Grade of bubbles %	38 zero	46 LBG	11 HBG	5 HBG+
	**Zero vs. LBG (38)**	**Zero vs. HBG**	**Zero vs. HBG+**	**Note**
**ANTHROPOMETRIC RISK FACTORS**				
Gender	–	–	–	Analysis of contingency did not show any gender related difference *p* = 0.40
Age (years)	*P* = 0.002	*P* < 0.0001	*P* < 0.0001	Age related bubble increase
Height (cm)	Ns	Ns	Ns	**NO** influence on bubbles
Weight (km)	Ns	Ns	Ns	**NO** influence on bubbles
BMI (kg/m^2^)	Ns	*P* = 0.01	*P* = 0.04	Increased BMI seems to increase bubbles
Fat mass (%)	0.012	0.0005	<0.0001	Increased of fat mass seems to increase bubbles
Lean body weight (%)	Ns	Ns	Ns	**NO** influence on bubbles
**DIVING RISK FACTORS**				
Depth	*P* = 0.048	*P* < 0.02	*P* < 0.001	Depth related bubble increase
Diving time	*P* = 0.02	Ns	Ns	**NO** relevant influence
GF	*P* = 0.0001	*P* = 0.0002	*P* < 0.0001	Increasing GF increase bubbles
Leading tissues	–	–	–	**NO** influence on bubbles *P* = 0.48 (analysis of contingency)
Minimum temperature	Ns	Ns	Ns	**NO** influence on bubbles

**Table:** 

**Other risk factors**	***P*****-value**	**Analysis of contingency**
Low visibility	0.0001	Low visibility reduces bubbles **BUT** by reduction of diving exposure *p* = 0.001
Workload	0.0003	Workload reduces high bubbles grade **BUT** by reduction of diving exposure *p* = 0.001
Environment	0.001	Diving in lake reduces high bubbles grade **BUT** by reduction of diving exposure *p* = 0.001
Gas used (Nitrox/Air)	0.90	**NO** influence on bubbles
Current	0.06	**NO** influence on bubbles
Perceived temperature	0.35	**NO** influence on bubbles
Suit	0.38	**NO** influence on bubbles
Feeling before diving rested/tired	0.13	**NO** influence on bubbles
Exercise before diving	0.06	**NO** influence on bubbles
Divers related problem	0.55	**NO** influence on bubbles
Equipment malfunction	**0.38**	**NO** influence on bubbles
Use of alcohol before diving	0.43	**NO** influence on bubbles

We found a statistically significant difference when comparing grade Zero vs. other grades in:

✓BMI lower in grade Zero vs. HBG (*p* = 0.02)
✓Fat mass was significantly lower in subjects with grade Zero vs. LBG (0.012), HBG (0.0005) and HBG+(<0.0001)
✓Age related difference was found lower in grade Zero vs. LBG (*p* = 0.01) than for HBG (*p* < 0.001) and HBG+ (*p* < 0.001)
✓Diving Exposure (see Table 2 for details)

The bubble grade showed no difference between fast tissues and medium tissue groups (*p* = 0.51); while slow tissues showed an increase in bubble grade when compared with both fast (*p* = 0.014) and medium (*p* = 0.01). However the slow tissues involvement regarded only 15 dives (one zero; 8 LBG; 5HBG; 1 HBG+).

Other risk factors seem linked with bubble formation but an in depth investigation showed that the effect was associated with an influence of diving exposure and consequently GF:

✓ Low visibility decreases High Bubble Grades (HBG and HBG+) (*p* = 0.002) but through significantly lower GF-values as compared to high visibility dives *P* < 0.001
✓ Intense workload during the dives seems to reduce High Bubble Grades (*p* = 0.001) most probably through a reduction of diving exposure

The other investigated risk factors did not show any significant relation with High Bubble Grades.

**Figure 1:**
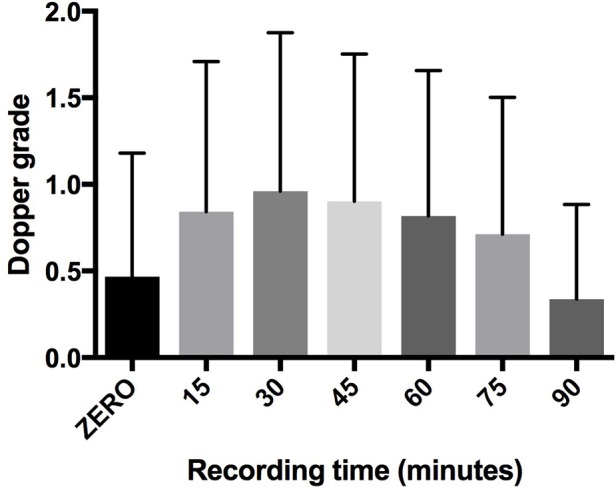
Trend of bubbles after dives. The peak of bubbles was localized around 30 and 45 min after dives: 90 min after dive the bubbles were significantly reduced.

Finally in the 448 dives for which Precordial Doppler recorded every 15 min we found that bubbles peaked between 30 and 45 min after the dive, irrespective of the bubble grade level (Figure 1).

### DCS risk factors (Table 3)

The 320 cases of DCS recorded in a specific section of the DAN Data base (all occurred in open circuit diving) were related to the analyzed factors such as:

✓ Higher percentage of female divers *P* < 0.0001
✓ Age significantly higher *p* < 0.001
✓ Less height, weight, and BMI *p* < 0.0001
✓ Higher percentage of fat mass *P* < 0.0001
✓ Lower lean body weight *P* < 0.0001
✓ Depth, dive time, and GF were statistically higher *p* < 0.0001, *p* = 0.001, *p* < 0.0001
✓ Lake diving *p* = 0.004
✓ Strong current and low visibility *p* < 0.0001 and *p* = 0.026
✓ Heavy exercise before diving *p* < 0.0001
✓ Heavy workload during diving *p* ≤ 0.0001

**Table 3:** Investigation on DCS Risk Factors.

	**Male**	**Female**	**Total**	
**Sample description**	*N* = 188 (59%)	*N* = 132 (41%)	*N* = 320	
	**DCS**	**Data base**	**Results**	**Note**
**ANTHROPOMETRIC RISK FACTORS**				
Gender	41.2% female	17% female	*P* < 0.0001	Females have higher possibility to develop DCS Notwithstanding similar bubble formation as compared to males
Age (years)	42 (23–67)	37 (10–82)	*P* < 0.0001	DCS increases when AGE increases
Height (cm)	173 (155–191)	178 (150–203)	*P* < 0.0001	
Weight (Km)	75 (49–120)	81 (40–125)	*P* < 0.0001	
BMI (Kg/m^2^)	24.5 (17–44)	25.6 (17–37)	*P* < 0.0001	Decrease of BMI seems to increase DCS
Fat mass (%)	34.05	23.7	*P* < 0.0001	Increased of fat mass seems to increase DCS
Lean body weight (%)	53.05	62.6	*P* < 0.0001	In DCS case we found a lower lean mass
Depth	32.4 (13–82)	27.1 (5–104)	*P* < 0.0001	Increase of depth Increases DCS
Diving Time	48.4 (17–104)	46 (10–130)	*P* = 0.001	Increase of time Increases DCS
GF	0.79 (0.4–1.1)	0.66 (0.05–1.2)	*P* < 0.0001	Increase of GF Increases DCS
Real minimum recorded temperature	23 (0.0–36)	17 (0.0–32)	*P* < 0.0001	Incidence of DCS increases when water temperature increases **BUT** In these cases warmer temperature increases diving exposure *P* < 0.0001
**OTHER RISK FACTORS**				
Environmental (Lake/Sea)	10.9%	6.7%	*P* = 0.005	Higher DCS incidence increases for lake dives **NOT** diving related *P* = 0.71
Presence of current	35.6%	24.8%	*P* < 0.0001	Incidence of DCS increases in presence of current **NOT** diving related *P* = 0.72
Low visibility	39.1%	33.3%	*P* < O.026	Incidence of DCS increases in low visibility **NOT** diving related *P* = 0.09
Physical exercise into 24 h before	90.3%	69.7%	*P* < 0.0001	Exercise before diving increases DCS **NOT** diving related *P* = 0.56
Workload (Intense)	86.6%	7.93%	*P* < 0.0001	Incidence of DCS increases in dives with high workload **NOT** diving related *P* = 0.62
Suit (Dry)	30.9%	19.0%	*P* < 0.0001	Incidence of DCS increases in dives with Dry suit **BUT** In these cases by an increase in diving exposure *P* = 0.0002
Thermal comfort (Confortable)	96.9%	94.02%	*P* = 0.08	**NO** influence on bubbles
Feeling before the dive (Rested or Tired)	93.4%	90.9%	*P* = 0.14	**NO** influence on bubbles
Divers related problem (No problem)	96.6%	94.4%	*P* = 0.96	**NO** influence on bubbles
Equipment malfunction	95.6%	97.2%	*P* = 0.08	**NO** influence on bubbles
No use of alcohol before diving	61.6%	57.6%	*P* = 0.17	**NO** influence on bubbles

*Some risk Factors increase the prevalence of DCS without any influence of bubble formation; these aspects could influence the effects of similar amount of bubbles*.

Other risk factors also appeared related to DCS but an in depth investigation showed that the effect was associated with increasing diving exposure and consequently GF:

✓ Higher water temperature *p* < 0.0001
✓ Dry Suit diving *p* < 0.0001

We did not find any other significant difference for all the other investigated risk factors (Table 3).

### In-depth analysis of GF-value in the 320 DCS cases (Table 4)

✓ Only eight cases (2.5%) showed a GF > 1
✓ 14 cases had a GF > 0.9 (4.4%)
✓ The majority of cases (236–73.7%) showed GF-values between 0.70 and 0.90
  * 37.5% between 0.8 and 0.9
  * 36.2% between 0.7 and 0.9
✓ 46 cases (14.4%) had a GF lower than 0.70
✓ 10 cases (3.4%) lower than 0.60
✓ Only 3 cases had a GF lower than 0.50

**Table 4:** GF in DCS cases.

**Numbers of cases**	**Percentage**	**GF**
8	2.5	>1
14	4.4	>0.9
120^*^	**37.5**	**>0.80**; **<0.90**
116^*^	**36.2**	**>0.70**; **<0.80**
46	14.4	<0.70
10	3.4	<0.60
3	0.94	<0.50

Only eight cases could be “predicted” by the model algorithm, all the other cases recorded in our DB would have been considered “undeserved.”

* *The majority of cases 236 (73.7%) presented GF values between 0.70 and 0.90*.

It is intriguing to note that all the eight cases that exceeded GF-value 1 involved the fast or the slow tissues, while no case of GF > 1 involved the medium tissues, indicating an apparent inability to correctly calculate and predict DCS by the current decompression models when medium HT compartments are involved as the leading tissue.

The distribution of DCS cases divided by single tissue is shown in Table 5.

Table 5Description of leading tissues grouping and their involvement in DCS.

**Table:** 

**Fast leading tissues group**	**Medium leading tissues group**	**Slow leading tissues group**
From 4 to 18.5 HT	From 27 to 38.3 HT	From 54.3 to 635 HT

**Table:** 

**Leading tissue**	**Mean maximum GF in DCS**	**Percentage of cases in DCS**	**Percentage of cases in DAN DB**
8	0.99	1.2	2.59
12.5	0.91	1.9	5.73
*18.5*	0.79	12.8	16.21
*27*	0.77	26.6	27.55
38.3	0.76	49.4	42.44
54.3	0.81	7.5	4.47
77	0.68	0.6	1.02
**SUMMARY LTG IN DCS VS. LTG DB (*P* = 0.0005)**			
Fast tissue	0.83	15.9	24.5
Medium tissue	0.72	75.9	70.0
Slow tissue	0.69	8.1	5.5
**STATISTICAL ANALYSIS OF LTG IN DCS VS. LTG DB**			
Fast tissues grouping	vs.	Medium tissues grouping	*P* = 0.0008
Fast tissues grouping	vs.	Slow tissues grouping	*P* = 0.0005
Medium tissues grouping	vs.	Slow tissues grouping	Ns

*Is appears that the algorithm can correctly predict inert gas accumulation only in the fast and slow compartments*.

*This is confirmed by the lower prevalence of fast tissue involvement in the DCS group than in the DAN DB, while the medium HT compartments were more significantly involved in the recorded DCS cases*.

Grouping the tissues into LTG we found:

✓ 75.9% of the DCS cases involved the Medium Tissues
✓ 15.9% the Fast tissues
✓ 8.1% the Slow tissues

This finding indicates that the prevalence of the different LTG was statistically different in the DCS group than in the total DB (*p* = 0.0005), in particular the DCS cases involved a lower percentage of “Fast Tissues” than expected.

A more in-depth statistical analysis considering the prevalence of the three groups separately showed:

✓ Prevalence of Fast tissues was statistically lower as compared to Medium tissues *p* = 0.0008
✓ Prevalence of Fast tissues was statistically lower as compared to Slow tissues *p* = 0.0005
✓ No difference in prevalence was found between medium and slow tissues *p* = 0.13 (Table 5)

## Discussion

The data collected by the DAN Europe Database have two important characteristics, in fact if on the one hand data recorded come from real-life dives, allowing for a “real picture” of the recreational diving community, on the other hand more than 11% of dives were performed during field research trips with an *ad hoc* research protocol allowing for accurate collection and in depth analysis of important variables, providing a large base of comparison to investigate Bubble and DCS related risk factors.

The DAN DB analysis shows that most dives were made in a “safe zone,” with an average depth of 27.1 m, average GF 0.66, and an average ascent speed lower than the currently recommended “safe” one. Even more importantly, very few deco omissions occurred; this indicates that divers tend to dive very conservatively.

Another interesting information is about the incidence of diver and equipment related problems which is reported to occur in only 6.3% of dives and that serious problems, fortunately, occurred only in a very limited fraction of these dives; for instance problems with breathing apparatus occurred only in 103 cases out of 39,099 dives, deco omission in just 20 dives and rapid ascent in only 109 dives. All together summing up to less than 0.6% of all recorded dives.

Our data confirm that the bubble peak occurs between 30 and 45 min after surfacing. This aspect is very important and indicates the importance to avoid efforts during this post-dive time interval, also considering that conditions increasing intrathoracic pressure, such as Valsalva maneuvers and physical efforts, can have negative implications for divers with Patent Foramen Ovale (Balestra et al., 1998).

But the main focus of this analysis was to investigate how certain risk factors may influence bubble formation (in particular high bubble grades) and DCS and the capacity to predict DCS trough the current decompression models, considering that in recent years diving medicine experts began to suspect that bubble formation and DCS occurrence could be linked not “only” to the dive profile but also to certain pre-dive conditions (Theunissen et al., 2013, 2015) and possibly to specific individual predisposition as already confirmed in a other diving related illnesses (Cialoni et al., 2015). The relation between bubble formation and DCS also seems to be more complex than previously believed and DCS in the presence of high bubble grades to be possibly influenced by other peripheral variables (Thom et al., 2015).

Our analysis showed little or no relation between bubble formation and many investigated “risk factors,” in fact only increased age and BMI appear to be related to increased bubble formation. It is interesting to note that height and weight separately did not appear to increase bubble formation, while their combined value (BMI) appeared to have a certain relation with higher bubble grades.

Because of this we included the analysis of fat mass, confirming a link with bubble formation, and apparently even more so when considering the DCS cases.

Although we could not find any really significant relation between the non-dive-profile related risk factors and bubbles it is intriguing to note that such risk factors, although not increasing bubble formation, appear to be related to DCS, allowing to infer that these risk factors may cause effects that, at similar bubble formation levels, can influence the diver's defense mechanism.

Such risk factors (current, low visibility, lake diving—usually cold and with very low visibility-, high workload during the dive) are all likely to cause a condition of stress. Therefore it is possible to hypothesize that humoral factors (including hormones) released in a stress condition can influence the effect of bubbles, and we have already started a more in-depth study about these possible variables.

A similar explanation could be used to understand why women are more subject to DCS even without marked (St. Leger Dowse et al., 2002; Lee et al., 2003) difference in bubble formation as compared to similar dives in men. As already claimed in the literature, different moments of the menstrual cycle can be considered as increasing the risk of DCS (Lee et al., 2003) in fact the DCS incidents were unevenly distributed throughout the cycle with the greatest percentage of incidents occurring in the first week of the menstrual cycle. Use of oral contraceptive pill (OCP) appeared to reduce the risk.

Another intriguing case is the effect of visibility on bubbles and DCS; our data in fact show that high visibility increases bubble formation (by an increase of depth, time, and GF facilitated by the good diving condition) but DCS prevalence is higher with low visibility. This also seems to indicate that even in the presence of lower bubble grades, the stress effect induced by low visibility, may increase deco-stress and bubble susceptibility.

Conversely (and somewhat more classically) it must be noted that some risk factors do indirectly cause an increase in bubble formation and DCS cases, by an increase in depth, diving time and GF facilitated by fair water temperature, dry suit use, and/or excellent visibility.

However, the most important data of our study come from the analysis of the 320 DCS cases. The most notable observation is that, although the analyzed dives implied inert gas saturation levels well within the currently adopted “safety limits,” the current decompression algorithms clearly show a very significant “gray area” in their ability to predict DCS, demanding further research and a more “patho-physiological” approach to decompression.

The majority of DCS cases recorded in our DB (73.7%) actually occurred in a GF-value range between 0.70 and 0.90, that is in an area where the diver has correctly followed the indications of the adopted decompression model, without any omission of safety stop, ascent rate etc.

Data showed that only eight out of 320 DCS cases showed a Gradient Factor >1, which means that only 2.5% of these cases would have been “predicted” by the utilized algorithm.

All the other cases would have been considered unpredictable, unexpected or, as they are now frequently defined, “undeserved.”

Furthermore, all the eight “deserved” DCS cases involved fast and slow tissues indicating a better capacity to predict an excess of saturation in these compartment as compared to medium tissues. This is conversely confirmed by the observation that the fast compartments were involved in the DCS cases in a lower percentage than their incidence as the lead compartment in the total DAN Data Base.

The majority of DCS cases that we analyzed actually involved medium HT tissues with computed inert gas super-saturation levels well below the “accepted” and “safe” *M*-values.

Considering the involvement of many biological and physiological parameters such as endothelial function (Theunissen et al., 2013, 2015), hydration (Gempp et al., 2009), vascular and lymphatic response (Hugon et al., 2009; Balestra, 2014), to mention only a few of the more recently studied variables, we believe that more research efforts are now necessary to further clarify these aspects of the complex pathophysiology of decompression.

We maintain that the reliability limit of the so far adopted dive computer validation protocols has been reached and that the new frontier is to further improve the ability to customize safe decompression limits according to physiological variables, be it pre-determined and based on available scientific evidence such as the data mentioned above or, in a foreseeable future, by a proper “diver-dive computer” interaction facilitated by real-time physiological sensor-assisted technologies. Furthermore the recent discovery of unexpectedly significant circulating bubbles in breath hold diving causing DCS (Cialoni et al., 2016) requires us to extend the DAN DSL DB also the Breath Hold Divers Community.

## Conclusion

In conclusion the first analysis of the DAN DB shows clearly that most dives were made in a Time and Depth “safe zone.” Interestingly certain risk factors appear to be related to DCS but not to significantly influence bubble formation, confirming that such risk factors may affect the individual response to similar bubble levels.

Our data also indicate that the current algorithms are well focused to predict the maximum allowed GF-value (and therefore the decompression risk) in fast compartments but are deficient in identifying the correct maximum GF in the medium compartments, which appear to be prevalent in the DCS cases analyzed in this study.

The DAN Europe DSL DB analysis can provide important data to improve recreational diving safety and this will further improve with the continuing entry of data in our DB allowing for an increasingly valid and complete data analysis.

## Ethics statement

All experimental procedures were conducted in accordance with the Declaration of Helsinki (World Medical Association, 2013) and were approved by the Academic Ethical Committee of Brussels (B200-2009-039). All methods and potential risks were explained in detail to the participants. All personal data were handled according to the Italian Law on privacy. Written informed consent was obtained from all the participants.

## Author contributions

DC: Contributions to the conception and design of the work, performed laboratory studies, contributions to the acquisition, analysis, or interpretation of data for the work; wrote the submitted manuscript. MP: Contributions to the conception and design of the work, performed laboratory studies, contributions to the acquisition, analysis, or interpretation of data for the work. CB: Contributions to the conception and design of the work, Oversaw the research program, reviewed the manuscript. AM: contributions to the conception and design of the work, Oversaw the research program, reviewed the manuscript.

### Conflict of interest statement

The authors declare that the research was conducted in the absence of any commercial or financial relationships that could be construed as a potential conflict of interest. The reviewer TM and handling Editor declared their shared affiliation.

## References

[B1] Baker E. C. (1998). Clearing up the confusion about deep stops, in Immersed - International Technical Diving Magazine, Vol. 3, Winter.

[B2] Balestra C. (2014). The lymphatic pathway for microbubbles. Diving Hyperb. Med. 44:1. 24687477

[B3] Balestra C. Germonpre P. Marroni A. (1998). Intrathoracic pressure changes after Valsalva strain and other maneuvers: implications for divers with patent foramen ovale. Undersea Hyperb. Med. 25, 171–174. 9789337

[B4] Balestra C. Theunissen S. Papadopoulou V. Le Mener C. Germonpré P. Guerrero F. (2016). Pre-dive whole-body vibration better reduces decompression-induced vascular gas emboli than oxygenation or a combination of both. Front. Physiol. 7:586 10.3389/fphys.2016.00586 27965591 PMC5127795

[B5] Bennett P. B. Elliott D. H. (1982). The Physiology and Medicine of Diving, 3rd Edn. London; Carson: Baillière Tindall; Published in the U.S.A, Canada by Best Pub.

[B6] Buhlmann A. A. (1982). [Experimental principles of risk-free decompression following hyperbaric exposure. 20 years of applied decompression research in Zurich]. Schweiz. Med. Wochenschr. 112, 48–59. 7071573

[B7] Cialoni D. Marabotti C. Sponsiello N. Pieri M. Balestra C. Lucchini V.. (2015). Genetic predisposition to breath-hold diving-induced hemoptysis: preliminary study. Undersea Hyperb. Med. 42, 75–83. 26094307

[B8] Cialoni D. Pieri M. Giunchi G. Sponsiello N. Lanzone A. M. Torcello L. (2016). Detection of venous gas emboli after repetitive breath-hold dives: case report. Undersea Hyperb. Med. 43, 449–455. 28763174

[B9] Deurenberg P. van der Kooy K. Leenen R. Weststrate J. A. Seidell J. C. (1991a). Sex and age specific prediction formulas for estimating body composition from bioelectrical impedance: a cross-validation study. Int. J. Obes. 15, 17–25. 2010255

[B10] Deurenberg P. Weststrate J. A. Seidell J. C. (1991b). Body mass index as a measure of body fatness: age- and sex-specific prediction formulas. Br. J. Nutr. 65, 105–114. 10.1079/BJN19910073 2043597

[B11] Eftedal O. S. Lydersen S. Brubakk A. O. (2007). The relationship between venous gas bubbles and adverse effects of decompression after air dives. Undersea Hyperb. Med. 34, 99–105. 17520861

[B12] Gempp E. Blatteau J. E. Pontier J. M. Balestra C. Louge P. (2009). Preventive effect of pre-dive hydration on bubble formation in divers. Br. J. Sports Med. 43, 224–228. 10.1136/bjsm.2007.043240 18308884

[B13] Hugon J. Barthelemy L. Rostain J. C. Gardette B. (2009). The pathway to drive decompression microbubbles from the tissues to the blood and the lymphatic system as a part of this transfer. Undersea Hyperb. Med. 36, 223–236. 20088241

[B14] James W. Waterlow J. C. (1976). Research on Obesity: A Report of the DHSS/MRC Group. London: H.M.S.O; U.K. Department of Health and Social Security/Medical Research Council Group on Obesity Research.

[B15] Lee V. St. Leger Dowse M. Edge C. Gunby A. Bryson P. (2003). Decompression sickness in women: a possible relationship with the menstrual cycle. Aviat. Space Environ. Med. 74, 1177–1182. 14620475

[B16] Marroni A. Bennett P. B. Cronje F. J. Cali-Corleo R. Germonpre P. Pieri M.. (2004). A deep stop during decompression from 82 fsw (25 m) significantly reduces bubbles and fast tissue gas tensions. Undersea Hyperb. Med. 31, 233–243. 15485086

[B17] Spencer M. P. Johanson D. C. (1974). Investigation of New Principles for Human Decompression Schedules using the Doppler Blood Bubble Detector. Office of Naval Research Tech Report. ONR Contract N00014-73-C-0094. Available online at: http://archive.rubicon-foundation.org/3788 (accessed December 2, 2015).

[B18] St. Leger Dowse M. Bryson P. Gunby A. Fife W. (2002). Comparative data from 2250 male and female sports divers: diving patterns and decompression sickness. Aviat. Space Environ. Med. 73, 743–749. 12182213

[B19] Theunissen S. Balestra C. Boutros A. De Bels D. Guerrero F. Germonpré P. (2015). The effect of pre-dive ingestion of dark chocolate on endothelial function after a scuba dive. Diving Hyperb. Med. 45, 4–9. 25964032

[B20] Theunissen S. Guerrero F. Sponsiello N. Cialoni D. Pieri M. Germonpré P.. (2013). Nitric oxide-related endothelial changes in breath-hold and scuba divers. Undersea Hyperb. Med. 40, 135–144. 23682545

[B21] Thom S. R. Bennett M. Banham N. D. Chin W. Blake D. F. Rosen A.. (2015). Association of microparticles and neutrophil activation with decompression sickness. J. Appl. Physiol. 119, 427–434. 10.1152/japplphysiol.00380.2015 26139218

[B22] Trout B. M. Caruso J. L. Nelson C. Denoble P. J. Nord D. A. Chimiak J. (2012). DAN Annual Diving Report−2015 Edition: A Report on 2010-2013 Data on Diving Fatalities, Injuries, and Incidents, ed Buzzacott P., Durham, NC. 26937540

[B23] Weathersby P. K. Hart B. L. Flynn E. T. Walker W. F. (1987). Role of oxygen in the production of human decompression sickness. J. Appl. Physiol. 63, 2380–2387. 3436872 10.1152/jappl.1987.63.6.2380

